# Teaching by Teleconference: A Model for Distance Medical Education across Two Continents

**DOI:** 10.4236/ojog.2015.513106

**Published:** 2015-11-18

**Authors:** Adeline Boatin, Joseph Ngonzi, Leslie Bradford, Blair Wylie, Annekathryn Goodman

**Affiliations:** 1 Department of Obstetrics and Gynecology, Massachusetts General Hospital, Harvard Medical School, Boston, USA; 2 Department of Obstetrics and Gynecology, Mbarara University of Science and Technology, Mbarara, Uganda; 3 Department of Obstetrics and Gynecology, University of Massachusetts, Worcester, USA

**Keywords:** Distance Learning, Videoconferencing, Global Health, Telehealth, Telemedicine

## Abstract

**Introduction:**

In Uganda, an estimated 120 obstetrician/gynecologists serve a population of 30 million people demonstrating the need to train additional skilled clinician leaders in reproductive health. In 2012, a partnership was formed with the Mbarara Regional Referral Hospital (MRRH) in southwest Uganda and the Massachusetts General Hospital (MGH) in Boston, USA, in part to increase access to specialist training. This report presents an update in the development of a teaching conference between the institutions.

**Methods:**

In June 2012, a didactic teleconference between the institutions was instituted. Various conferencing tools were tried: direct telephone connection, Ventrilo™ conferencing system and Skype™ via personal computer or smart phone. In Mbarara, Internet was accessed via cellular data. In Boston, Internet was accessed via hospital network or cellular data. All lectures were HIPAA compliant. PowerPoint lectures were stored in a collective Dropbox™ that could be accessed and downloaded prior to lecture dates.

**Results:**

Over 30 months, 30 lectures were given. Lecturers included faculty and fellows from maternal fetal medicine, gynecology oncology, urogynecology, family planning, psychiatry and obstetric anesthesia. A patient case pertinent to the teaching topic framed the discussion. About 20 participants attended each lecture. Internet connectivity was the biggest challenge. Ultimately audio Skype via cellular data proved the most successful modality and became the method of choice.

**Conclusion:**

A successful collaboration in medical education via teleconference is sustainable, low cost, and beneficial to both resource-rich and resource-poor institutions. Expertise can be shared bilaterally and internationally by individuals potentially unable travel.

## 1. Introduction

In the last two decades there has been a fundamental shift in global connectivity and awareness. Parallel to this shift, and likely fuelled by increased connectivity, there has also been a marked growth in global health programs and initiatives, in particular an expansion of academic partnerships between high and low-income countries. These have emerged to fulfill dual needs; the desire and interest for students and faculty from higher income countries to have exposure to some of the conditions and diseases more prevalent in low income countries and a mutual desire to use the resources and expertise available in academic institutions to reduce some of the stark disparities in health care outcomes seen globally.

Addressing the disparity in the availability of medical specialists is often a major component of such partnerships. The World Health Organization estimates a shortfall of 4.3 million medical providers globally, with the deficit overwhelmingly concentrated in low income countries [1]. Stark disparities also exist in access to physicians with specialty and sub-specialty training; only 12% of the world’s specialist surgical workforce, including surgeons, anesthesiologists and obstetrician gynecologists reside in sub-Saharan Africa, where over a third of the world’s population lives [2]. This shortfall in providers not only compromises current access to care in areas with deficits, but also impacts the ability to continue and expand future access by making it extremely challenging to train the next generation of providers. A sole obstetrician-gynecologist responsible for thousands of women and attending to one complication after another will be hard pressed to find the time and, perhaps mental energy required to provide quality training to their junior or assistants. Relying on the apprenticeship model historically employed by surgical specialties will thus be insufficient to expand the workforce to the numbers required for safe access.

Technology is now an established component of health care provision and training. Distance learning or tele-learning has been used widely to expand access to medical education. Teleconferencing for education has been defined as using real-time and live programming with participants at two or more sites [3]. Most published experience with such distance education programs is limited to participants at remote sites within the same country [4]–[7]. Using distance learning to facilitate education between countries and across academic partnerships is a relatively new use, though results have been promising [8].

In this report we present our experience in building a low cost teleconference as a way to facilitate the ability of an academic partnership to expand access to sub-specialty obstetrics and gynecology training.

## 2. Methods

### 2.1. The Partnership

In 2012 an academic partnership was formed between the Departments of Obstetrics and Gynecology (OB/GYN) at the Mbarara Regional Referral Hospital (MRRH) in Mbarara, Uganda, and the Massachusetts General Hospital (MGH) in Boston, USA. Both institutions are referral centers and provide tertiary level obstetric and gynecologic care to a large surrounding population. The goals of this partnership were to foster bilateral education of residents in both departments, thus increasing capacity, and to increase the quality of care provision and promote research.

With only 10 faculty and no fellowship-trained subspecialists, the MRRH Department of OB/GYN faces the challenge of increasing capacity and depth of knowledge amongst its faculty and residents without easy access to sub-specialty obstetric and gynecologic training and expertise. A needs assessment conducted early in our partnership identified this educational gap as a key area of need. Through several face-to-face meetings, observation of clinical rounds and didactics, the concept of a teleconference evolved as a low cost strategy to facilitate distance learning for MRRH residents and to promote interaction between trainees at both institutions. The specific objectives of the teleconference were to 1) increase the breadth and depth of the didactic portion of bilateral resident education through case-based discussion 2) spur interaction with clinicians from different training and work environments to prompt new insights into the management of disease and systems of care across both institutions and 3) demonstrate that teleconferencing is an effective teaching tool for members of both a local and remote audience.

### 2.2. Building the Teleconference Program

Though the initial conception of the program occurred with face-to-face meetings of involved partners, implementation and execution largely occurred electronically. Email communication was used to plan the curriculum and select monthly teaching topics. To encourage bilateral involvement each lecture had speakers from both institutions. Typically a case presentation relevant to the selected topic was prepared and presented by a resident from MRRH. The didactic portion to the lecture was prepared and presented by fellows in training or faculty from MGH. The lectures lasted 60 minutes. The content of the lectures is listed in Table 1. Each lecture started with a case report, was followed by a didactic lecture, and finished with a 15-minute discussion period. All ten Ugandan faculty participated in the teleconferences. The ages of the participants ranged from 21 to 60 years of age. The genders of the participants were evenly divided between men and women. The educational level of the participants ranged from being in medical school, in the obstetrics and gynecology residency program, or on medical faculty of the respective Boston and Ugandan departments of Obstetrics and Gynecology. PowerPoint lectures were HIPAA compliant and prepared ahead of time so they could be shared via email or cloud storage (Dropbox™) for personal access and download. These lectures were sent ahead of scheduled teleconferences. The effectiveness of the conference was evaluated through email feedback.

Teleconferences were planned to recur every third Tuesday of the month at 7 am Eastern Standard Time (EST) and 2 or 3 pm Eastern African Time (EAT) depending on daylight savings in the US. Communication via web conferencing was the primary mode of teleconferencing. Each planned conference would begin with an international phone call between organizers on each end to establish contact prior to web conferencing.

Web conferencing tools used included Ventrilo™ and Skype™, through a number of interfaces: personal and hospital computer and smart phones. An international call using mobile phone was available as a back up if Internet connectivity failed. In Boston, free Wi-Fi connections were available through network systems. At times personal cellular data networks were used though such use incurred no additional cost to the end user. In Mbarara, no Wi-Fi networks were available. Internet access was obtained by purchasing cellular data through established commercial networks. On average 1 GB of data costs USD12. Approximately 100 MB and 300 – 500 MB of data is typically required for an hour of continuous audio and video web-conferencing respectively. In Boston, lectures were displayed to residents on projector equipment on site at MGH. In Mbarara, similar projection of lectures was performed and audio or video from the web conference provided through personal laptop computer (PC). In the event of electricity outages in Mbarara, slides were viewed via personal computer.

## 3. Funding

Funding for the project came from an endowed women’s healthcare global health fund held in the Department of Obstetrics and Gynecology at MGH.

## 4. Results

From June 2012 through January 2015, thirty teleconferenced lectures were planned and given (Table 1 and Table 2). The conferences were progressively streamlined using the email feedback.

**Figure 1:**
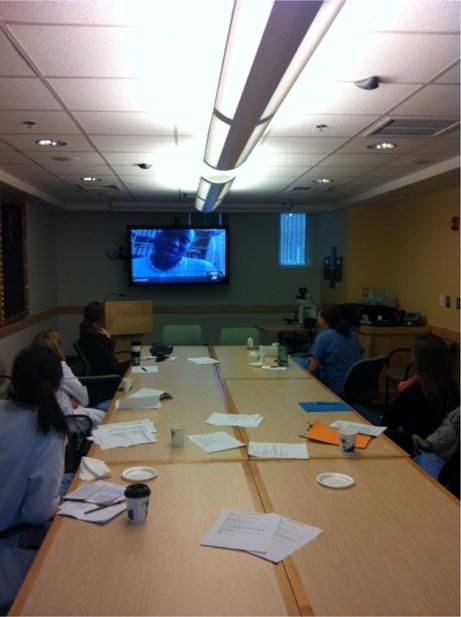
Teleconference setting in Boston

**Figure 2:**
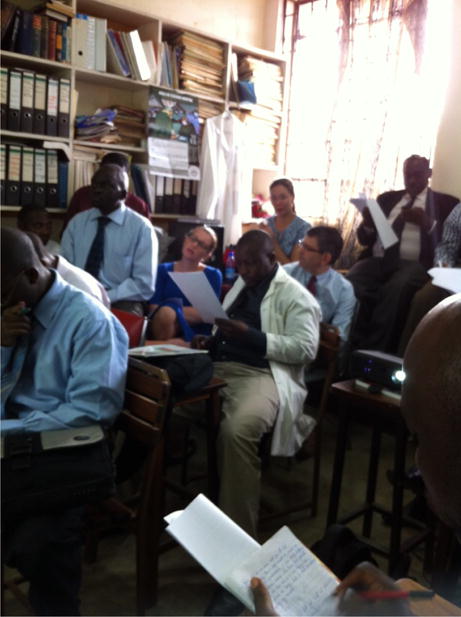
Teleconference in Mbarara, Uganda.

Topics covered the breadth of obstetrics and gynecology with some emphasis given to sub-specialty areas of gynecology oncology and maternal fetal medicine given the demonstrated need. There were an average of twelve attendees at MGH (students, residents, fellows, and faculty) and 20 at Mbarara (Figure 1 and Figure 2). The meetings were unrestricted and open to all members of the Boston and Mbarara departments. Though primarily targeted to residents, at times other members of the care team including midwives, nurses and nursing students attended particularly for lectures aimed at strengthening teams and providing overarching care principles.

Internet connectivity was the biggest challenge to successfully sharing lectures bilaterally. Table 2 summarizes the connectivity challenges and outcomes for the first 18 months of conferencing. Problems in connectivity were mostly commonly manifested as delayed transmission of audio or video, freezing of transmission and dropping of connections requiring redialing and reconnection. Five of the nineteen lectures were given without interruption. Skype™ became the modality of choice for web conference. Ventrilo™ was dropped after several lectures because of the difficulty in real time conversations. Disruptions in Internet connectivity occurred from access at both institutions. Early in the development of the program we transitioned from hospital Wi-Fi networks at MGH, which proved unreliable, to more robust personal cellular data networks accessed via Smartphone. In Mbarara, cellular data networks worked well for the most part, but also contributed to interruptions as described above. Initial attempts at including video conferencing led to many interruptions in connectivity, thus this was also eliminated early in the development of the program and audio alone relied upon for conferencing.

## 5. Discussion

Meeting the health demands of a population requires access to skilled, well-trained health providers. Current shortages in such skilled providers calls for multiple and innovative strategies to increase the work force and in particular to increase the numbers of providers with specialized training. We present a simple and low cost model for education that harnesses academic partnerships and easily available resources to promote bilateral resident education and increase access to sub-specialty expertise in settings typically excluded. Our experience adds to modest literature examining distance education across countries and provides encouraging preliminary findings for the development and expansion of such programs.

Commitment from both partner institutions was critical for this program to develop and become sustainable. An identifiable advocate from each institution with the responsibility of facilitating communication was extremely important in the planning of lectures and execution of teleconferences. We also found that during the lecture having faculty familiar with the environment and culture of both settings played an important role in facilitating discussion. This was particular relevant when presenting fellows or faculty from MGH had no familiarity with the clinical environment at MRRH. It was also helpful for cultural reasons, for example to facilitate the translation of used idioms, language patterns and even accents specific to each geographical setting.

Internet connectivity remained the biggest challenge to successful conferencing though relatively few-six of thirty lectures, had to be cancelled due to failed connections or technical issues. Relying on cellular data at both institutions and eliminating video dramatically improved the quality of connection. In such partnerships it is often assumed that infrastructure at the low-income institution will be the limiting factor; therefore it was an important lesson to consider connectivity issues at MGH and actively address those. Advance planning and sharing of lecture materials provided both sides with the tools to proceed as a back up in instances of failed Internet connectivity. This was important to ensure that information exchange still occurred and facilitated separate learning in those few instances when web conferencing was unsuccessful.

The ability to have real-time interaction and discussion is a major attraction for distance learning programs based on teleconferencing over e-learning and web-based learning that is self directed or asynchronous. Videoconferencing greatly facilitates this, and yet early in our program we had to eliminate this due to constraints in bandwidth speed. We found that audio conferencing did continue to provide some level of interaction, however, face-to-face interaction would likely have stimulated more interaction between groups of residents and faculty who have never met in person. Requiring case presentations from residents at the distance site-MRRH became an important method to deliberately encourage active participation from both sites. Locally chosen case presentations prompted more interaction from those residents and required faculty and fellow from MGH to consider differences in clinical environment that may be present. This not only facilitated bilateral engagement but also provided the platform for bilateral learning opportunities and discussion. Introducing other deliberate strategies such as technology based user participation may further increase interaction and learning opportunities.

Video-conferencing has been performed successfully in other examples of distance learning though with higher costs. As a voluntary program with limited funding we sought to introduce this program with little to no costs. We achieved this on a budget of under $500. Current available methods for data transmission during teleconferencing are satellite communication, Internet Protocol (IP), Integrated Services Digital Network (ISDN) and cellular networks [3]. Satellite communication costs tend to be prohibitive, with start-up costs averaging USD 30,000 and rental costs of USD 500 – 1000/month. ISDN requires access to public switched telephone networks over wired telephone systems. These are currently unavailable in many areas of low-income countries and with current widespread use of mobile phones unlikely to be applied. IP and cellular networks are therefore most relevant for use in this type of program.

We relied on Skype™ as free software available to facilitate conferencing via IP and cellular networks. This was used with similar success in a distance education program facilitating access to anesthesia subspecialty education [8]. Ventrillo was abandoned because it required PCs on both sides and required holding down the keyboard key to talk. This led to confusion when there was a question from the other side. Other distance learning programs have had success using alternative software, of which Polycom^®^, has been most commonly described [4] [5] [9] [10]. Costs for using this software average 2 million USD for a large corporation (http://www.bradreese.com/blog/polycom-6-8-2010.htm).

A future and important step in our program will be to evaluate the effectiveness and acceptability of the teleconference to residents at both institutions. Evidence from other programs is encouraging with high acceptability and comparable efficacy for students both on site and off site [5] [9]–[13]. This evidence is limited to programs that offer distance learning within the same country and often with participants from the same institutions at different sites. Evaluations of programs such as ours, where there are substantial differences in culture, clinical environments and across much larger distances will be important to determine if findings will be similar.

## 6. Conclusion

In summary, despite some challenges in connectivity we were able to develop and sustain a distance education program across two continents and two institutions for over two years at little cost. This program facilitated access to subspecialty education for residents in-training in a country where such access has been unavailable. It also provided the opportunity to spur interaction and clinical discussion among faculty, fellows, and residents from dramatically different clinical environments creating bilateral learning opportunities.

## Figures and Tables

**Table 1:** Lecture topics.

***Gynecology Oncology***
Loop Electricalsurgical Excisiion Procedure (LEEP) versus cryotherapy
Cervical dysplasia
Cervical cancer staging
Basic principles of radiation oncology
Basics of Colposcopy
Human Papilloma Virus (HPV)-related diseases
HPV and HPV vaccination
Palliative care in low resource countries
Management of a complex adnexal mass
***Maternal Fetal Medicine***
Electronic fetal monitoring
Meconium
Malaria in pregnancy
Fetal Monitoring
Management of Psychiatric Disease in Pregnancy
***General Obstetrics and Gynecology***
Surgical Anatomy
Management of ectopic pregnancy Management of postpartum hemorrhage
Vulvar diseases
Laparoscopic surgery
Management of incomplete abortion
***Urogyencology***
Management of vesicovaginal fistula
***Other***
Basic Obstetric Anesthesiology
Building Stronger Teams in OB/GYN
Simulation for Medical Education and Critical Care

**Table 2:** Boston to Mbarara teleconferences: technology and challenges.

Date	Connection	Device interface		Problem	Outcome

		MGH	MRRH		
06/2012	Skype	Hospital PC	Personal PC	None	No interruptions-full lecture
07/2012	Skype	Hospital PC	Personal PC	Uganda Internet	Interruptions-partial lecture
08/2012	Skype	Personal PC	Personal PC	Uganda Internet	Interruptions-full lecture
09/2012	Ventrillo	Hospital PC	Personal PC	None	No interruptions-Full lecture
10/2012	Ventrillo	Hospital PC	Personal PC	None	No interruptions-Full lecture
11/2012	Ventrillo	Hospital PC	Personal PC	Unable to connect	Separate lectures given
12/2012	Skype	Smartphone	Personal PC	None	Interruptions-full lecture
01/2013	Skype	Smartphone	Personal PC	Internet connectivity	Interruptions-full lecture
02/2013	Skype	Smartphone	Personal PC	Internet connectivity	Interruptions-full lecture
03/2013	Skype	Smartphone	Personal PC	Internet connectivity	Interruptions-full lecture
04/2013	Skype	Personal PC	Personal PC	Internet connectivity	Interruptions-full lecture
05/2013	Skype	Personal PC	Personal PC	None	Interruptions-full lecture
06/2013	Skype	Smartphone	Personal PC	Internet connectivity	Interruptions-full lecture
07/2013	Skype	Smartphone	Personal PC	Internet connectivity	Interruptions-full lecture
08/2013	Skype	Smartphone	Personal PC	Internet connectivity	Separate lectures
09/2013	Skype	Smartphone	Personal PC	Internet connectivity	Interruptions-full lecture
10/2013	Skype	Smartphone	Personal PC	Internet connectivity	Separate lectures
11/2013	Skype	Smartphone	Personal PC	Internet connectivity	Interruptions-full lecture
12/2013	Skype	Smartphone	Personal PC	Internet connectivity	Interruptions-full lecture
1/2014	Skype	Smartphone	Personal PC	none	Interruptions-full lecture
2/2014	Skype	Smartphone	Personal PC	Internet connectivity	Separate lectures
4/2014	Skype	Smartphone	Personal PC	Unable to connect	Separate lectures
5/2014	Skype	Smartphone	Personal PC	None	No interruptions-full lecture
6/2014	Skype	Smartphone	Personal PC	Internet connectivity	Interruptions-full lecture
7/2014	Skype	Smartphone	Personal PC	None	No interruptions-full lecture
9/2014	Skype	Smartphone	Personal PC	None	No interruptions-full lecture
10/2014	Skype	Smartphone	Personal PC	None	No interruptions-full lecture
11/2014	Skype	Smartphone	Personal PC	None	No interruptions-full lecture
12/2014	Skype	Smartphone	Personal PC	Internet connectivity	Interruptions-full lecture
1/2015	Skype	Smartphone	Personal PC	None	No interruptions-full lecture
